# Statin cost effectiveness in primary prevention: A systematic review of the recent cost-effectiveness literature in the United States

**DOI:** 10.1186/1756-0500-5-373

**Published:** 2012-07-24

**Authors:** Aaron P Mitchell, Ross J Simpson

**Affiliations:** 1 Duke University Medical Center, Medical Residency Office Rm 8254DN, 2301 Erwin Rd, Durham, NC, 27710, USA; 2 Department of Cardiology, UNC-Chapel Hill School of Medicine, 101 Manning Drive, Chapel Hill, NC, 27514, USA

**Keywords:** Statin, Cost-effectiveness, Primary prevention, Drug cost, Systematic review

## Abstract

**Background:**

The literature on the cost-effectiveness of statin drugs in primary prevention of coronary heart disease is complex. The objective of this study is to compare the disparate results of recent cost-effectiveness analyses of statins.

**Findings:**

We conducted a systematic review of the literature on statin cost-effectiveness. The four studies that met inclusion criteria reported varying conclusions about the cost-effectiveness of statin treatment, without a clear consensus as to whether statins are cost-effective for primary prevention. However, after accounting for each study’s assumptions about statin costs, we found substantial agreement among the studies. Studies that assumed statins to be more expensive found them to be less cost-effective, and vice-versa. Furthermore, treatment of low-risk groups became cost-effective as statins became less expensive.

**Conclusions:**

Drug price is the primary determinant of statin cost-effectiveness within a given risk group. As more statin drugs become generic, patients at low risk for coronary disease may be treated cost-effectively. Though many factors must be weighed in any medical decision, from a cost-effectiveness perspective, statins may now be considered an appropriate therapy for many patients at low risk for heart disease.

## Findings

### Background

The HMG-CoA reductase inhibitors, or “statins,” are proven in multiple randomized, controlled, clinical trials to lower cardiac morbidity and mortality [1]. Statins successfully lower LDL cholesterol in most patients, with substantial reductions in the risk of major coronary events, such as MI [2], and stroke [3,4]. Statins reduce mortality in patients with pre-existing coronary disease [1], but it is unclear if this mortality benefit holds for primary prevention [3,5]. Statins are generally well-tolerated and have a low rate of major side effects [6].

Statin trials suggest that the relative risk reduction of cardiac disease is constant regardless of each patient’s overall risk [1,7,8]. Therefore, the number needed to treat is lower in higher-risk groups; more heart attacks will be prevented by treating 100 patients at high risk for disease than at low risk. Accordingly, statins are more cost-effective in higher-risk groups, since fewer patients must be treated for each event prevented [9]. Cost-effectiveness analyses (CEAs) have generally found that statins are cost-effective in secondary prevention, since patients with established heart disease are at the greatest risk [10,11]. However, it remains unclear whether statins are cost-effective when treating healthy patients without known cardiac disease.

The question of statin use in primary prevention remains unresolved in the cost-effectiveness literature. A meta-analysis of CEAs of statins published before 2002 concluded that patients’ cardiac risk was the primary determinant of whether statin treatment was cost-effective, and that it was unlikely that statins would be cost-effective for patients with an annual risk of <1% [9]. Conversely, a 2003 review found that statins would likely be cost-effective for primary prevention in groups without known cardiac disease but with cardiac risk factors [11]. However, since these studies were published, the price of statins has fallen substantially and will likely continue to decrease [12], a trend which would be expected to improve the cost-effectiveness of statins. The apparent lack of consensus in the literature and the changing price of statins may create confusion about whether it is economical to use statins for lower risk patients.

To address this question in the current US market, we conducted a systematic review of the literature to determine when statin therapy becomes cost-effective for low risk patients.

### Methods

**Figure 1:**
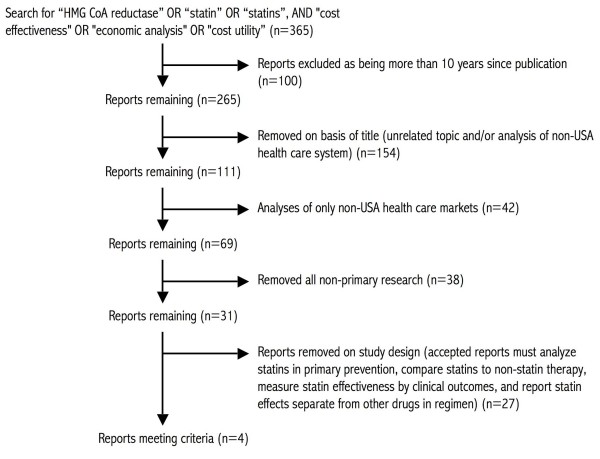
**Article selection**. Flowchart of article selection.

We conducted a systematic review of the literature of CEAs of statins (Figure1). Published studies were identified by a Pubmed search using the criteria (“HMG CoA reductase” OR “statin” OR “statins)” AND (“cost effectiveness” OR “economic analysis” OR “cost utility)”; the search was last conducted on January 2, 2011. This search yielded 365 results. Papers on unrelated topics or those without primary research were removed. Further inclusion criteria included: publication since the year 2000, analysis of health costs in the USA, evaluation of statins in primary as opposed to secondary prevention, measurement of statin effectiveness by cardiac endpoint reduction rather than cholesterol lowering, and comparison of statins to non-statin therapy (either placebo or non-statin active therapy). Five studies remained. One additional study was excluded because it analyzed the effectiveness of whole drug regimens but not of statins in isolation, leaving four studies for analysis [12-15].

For each study, we determined 1) the presumed price of statins that each study used in its analysis, and 2) the resulting incremental cost-effectiveness ratio when treating patients at multiple Framingham risk levels. As each paper presented data in a different format, the details of our data extraction are found in the Additional file 1.

Drug prices and incremental cost-effectiveness ratios (ICERs) [16] were converted to 2006 dollars using the consumer price index for medical care. The ICERs reported by each study were stratified by Framingham 10-year risk of a major coronary event. For each risk group, ICERs were then expressed as a function of the drug price. Some studies provided results for a variety of drug prices, or provided enough data for their results to be extrapolated to different drug prices; where possible, we used these different drug price-scenarios as additional data points in our analysis.

### Results

Four cost-effectiveness analyses of statin therapy for primary prevention met inclusion criteria (Table1). All studies analyzed health care costs from the health care system perspective – inclusive of and limited to all direct medical costs to all payers resulting from the statin therapy. The time horizon varied from as little as 5 years to lifetime. Annual drug prices varied from $770 to over $1,500 in 2006 dollars. Models of drug effectiveness were based on clinical trials of pravastatin in three of the four studies, and in one on “low intensity” and “high intensity” statin regimens [12]. Two studies segregated men and women in their analysis, while one analyzed men only and one studied all persons in aggregate. Studies compared statins to placebo [13], diet modification [14], aspirin [15], or to other statin treatment models [12]. Three of the studies assessed cost-effectiveness in terms of quality-adjusted life year (QALY) gained, and one measured life years gained (LYG) [13]. 

**Table 1:** Source studies

**Study, 1**^**st**^** Author**	**Year**	**Population**	**Drug Studied**	**Time Horizon**	**Outcome Measured**
Prosser LA	2000	Age 35-84, LDL >159	Pravastatin	30 years	QALY
Caro JJ	2003	Age >45	Pravastatin	5 years	LYG
Pignone M	2006	45-year old men	Pravastatin	Lifetime	QALY
Pletcher MJ	2009	Age >35	All statins	30 years	QALY

Methodological differences among studies. QALY, quality adjusted life year; LYG, life year gained; LDL, low density lipoprotein cholesterol.

**Figure 2:**
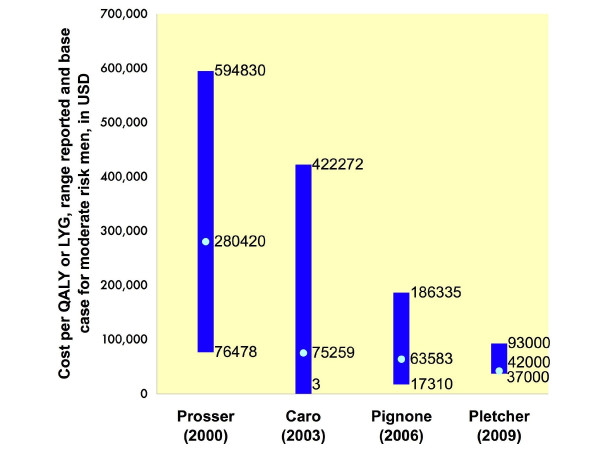
**Reported ranges of cost-effectiveness ratios.** Reported range of cost per QALY or LYG for the use of statins for primary prevention within each study. Also shown is the projected cost per QALY or LYG for men at average or 7.5% 10-year risk for coronary disease. Values shown are derived from the “base case” assumptions of the model used in each study, with the variable being the risk level of the patient population or the treatment strategy applied. For Prosser et al., Caro et al., and Pignone et al., these values represent ranges for men at varying risk levels. For Pletcher et al., these values represent the cost-effectiveness of statin treatment strategies as compared to a baseline of standard primary prevention guidelines. All values adjusted to 2006 dollars. QALY, quality adjusted life year; LYG, life year gained.

The studies offered varying assessments of the cost-effectiveness of statins (Figure2). Costs ranged from as much as $590,000 to as little as $3 to extend life by one year. The more recent studies found statin therapy more likely to be cost-effective.

**Figure 3:**
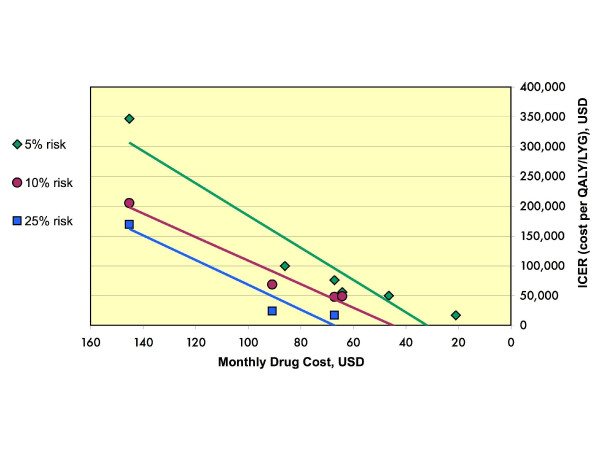
**Cost-effectiveness ratios for patients at specific risk levels.** Incremental cost-effectiveness ratios, shown on the y-axis, were extracted from each article and grouped by 10-year Framingham cardiac risk levels. Within each risk level, the cost-effectiveness of statin treatment was compared with the presumed annual price of statin drugs from each study. Linear regression analysis using Excel spreadsheet function is also shown. QALY, quality adjusted life year; LYG, life year gained.

To assess whether these differing conclusions were the result of the studies’ having modeled patients at different cardiac risk levels, we compared ICERs *within* risk groups. This resulted in greater consistency among the source studies for patients at 5%, 10%, and 25% 10-year risk for a major cardiac event by Framingham score. Once the varying levels of cardiac risk had been controlled for, the primary factor driving the cost-effectiveness of statins was the presumed drug price (Figure3). There was a direct relationship between the cost of statins and each study’s conclusions. The cheaper the statin, the lower its associated ICER. The correlation between drug price and cost-effectiveness was strong across the different studies. Within each risk group, the cost profile improved as the drug price fell.

We confirmed that statin cost-effectiveness increases with the risk level of the population. Using the $50,000 per QALY threshold, treatment of patients at relatively higher risk (25% over 10 years) was cost-effective at higher drug prices than for patients at lower risk. Additionally, it became cost effective to treat patients with a 10% 10 year risk at statin prices under $843.27 per year (approximately $70 per month), and even patients with a 5% 10 year risk at prices under $604.72 per year (approximately $50 per month).

### Discussion

Within the United States health care system, we found a strong relationship between drug price and the incremental cost-effectiveness ratio for statin therapy. Our results support the conclusion that drug cost is the primary determinant of statin cost-effectiveness.

Our findings are important as they suggest that there is substantial agreement in the medical literature on statin cost-effectiveness. The apparently large, unreconciled discrepancies of previous reports may have made it difficult for clinicians to draw conclusions about statin cost-effectiveness, especially for lower risk patients. For example, other reviews of statin CEAs found reports of statin costs ranging from $6,500 to $490,000 per LYG in low-risk patients [9]. With reported ICERs varying by orders of magnitude, these results seemingly indicate a lack of consensus on the cost-effectiveness of these drugs.

Our analysis suggests a resolution of this dilemma. We demonstrate that after controlling for patient risk level and drug cost, these studies appear to agree. This consistency is supported by one author who noted that the differences between his findings and those of an earlier study [14] may have been the result of “lower estimates for the pill costs of statins” [12]. Rather than the wide range of conclusions apparent at first glance (Figure2), it seems that there is agreement in the cost-effectiveness literature on statins.

Our results have implications for the use of statins in clinical practice. As statin ICERs improve as drug prices fall, from a cost perspective it will eventually become appropriate to treat very low-risk patients. For example, under ATP-III guidelines, a healthy individual with a 10-year risk for heart attack or cardiac death of 5% would not receive pharmacologic treatment unless his LDL exceeded 190 g/dL, whereas our results imply that it would be cost-effective to treat this patient if statins could be obtained for less than $604.72 per year. Our conclusions are consistent with those of Pletcher et al., that “with lower costs, extending statin therapy to larger, lower-risk segments of the U.S. population becomes cost-effective [12].” From a cost perspective, treatment decisions should not be made categorically based on strict criteria, but by an evaluation of each patient’s risk level as well as the current drug availability. It should be noted that treating patients based purely on an evaluation of cost-effectiveness would mean extending statin therapy beyond current treatment guidelines.

If drug costs are part of the treatment calculus, then health care providers need to be familiar with the prevailing prices in their communities. Where in this spectrum of high costs and targeted treatment vs. low costs and extended treatment do we currently lie? Are the low drug prices necessary to treat a 5%-risk patient cost-effectively still far in the future, or have they already come to pass? Pletcher et al., published in 2006, estimated the annual cost for low-intensity statins at $770 [12]. Since then, prices for generic simvastatin have already fallen by 60% [17]; our results predict that at this price it would be cost-effective to treat patients at 5% 10-year risk. Others have found that at prices below $0.10 per pill, it becomes not only cost-effective but cost-*saving* to treat adults at all risk levels [12]; the available prices at some large retailers in the USA have already passed this threshold [18]. At these low prices, our analysis supports the conclusion that it is cost-effective to treat more Americans with statins than would qualify for pharmacologic cholesterol reduction under current guidelines.

Demographically, what does a patient at a 5% Framingham risk level look like? As an example, any male smoker over the age of 50, even one with favorable blood pressure and lipid profiles, has a 10-year risk of greater than 5%. Furthermore, even the lowest-risk male over age 60 exceeds the 5% risk level [19]. Our results indicate that it may be cost-effective to treat both of these hypothetical patients with statins.

### Limitations

Our study pertains only to primary prevention: patients with no history of coronary disease. Indeed, statins are far *more* cost-effective in secondary prevention, and the benefits of their usage in this context have been well-established in the cost-effectiveness literature [12]. It would be a mistake to withhold statin therapy from a post-MI patient, for example, because statins were not obtainable at the prices discussed in this study, as our incremental cost-effectiveness ratios are applicable only to primary prevention.

Importantly, each study we reviewed assessed only the direct medical costs of patient care. While this is standard practice for cost-effectiveness analyses, it excludes secondary economic costs to society such as losses in worker productivity. These costs can potentially be very large, and when they are factored into the analysis statins become more cost-effective, and possibly cost-saving, across many scenarios [20].

Nevertheless, the expanded use of statins in the primary prevention setting would be expected to increase overall health care costs. Use of these drugs in additional low-risk patients may be, as our analysis suggests, a cost-effective intervention; however, this would require the dedication of significant health care resources [10].

Head-to-head comparison of individual statin drugs was beyond the scope of our study. It was assumed that the benefits of statins are proportional to their lipid-lowering effects, and therefore that the same cholesterol reduction obtained by different doses of different agents will yield similar results. As statins currently vary widely in price, it is to be expected that different degrees of lipid lowering (and consequently different degrees of cardiac protection) would be achievable with the same health care dollar depending upon which agent is chosen. This is an assumption, however, and the fact that 3 of the 4 studies based their models on data derived from pravastatin trials may limit the generalizability of our findings to other statins.

The most important assumption of our sources is that the relative risk reduction achieved with statins is constant across primary prevention subgroups, including low risk patients. This assumption is not universally accepted [10]. In addition, our source studies assumed statins to be safe, with negligible costs associated with any rare, adverse drug reactions. One source study explicitly included the health care costs of statin-associated adverse events such as myopathy, and concluded that these events are rare enough to have a negligible effect on incremental cost-effectiveness ratios [15]. Based on the available clinical trial and epidemiologic literature, we believe these assumptions are valid.

Our conclusions are not affected by a recent Cochrane meta-analysis emphasizing “caution” in the use of statins in primary prevention [21]. This meta-analysis agreed with previously reported estimates of the benefits of statins in primary prevention – an approximately 30% reduction in combined fatal and non-fatal cardiovascular events. Since the studies we analyzed herein used similar estimates in generating their cost-effectiveness data, their results are in line with the statin benefits outlined by the Cochrane meta-analysis. This meta-analysis also found no increased rate of any adverse event in patients receiving statin therapy for primary prevention, including myalgia, rhabdomyolysis, or cancer, supporting the supposition that the management of adverse events has little, if any, effect on statin cost-effectiveness. Regardless, the recommendation for caution was due to a concern that adverse events may have been under-reported in statin clinical trials.

Our conclusions differ from those of a recent cost-effectiveness analysis [22]. Its results suggest poor statin cost-effectiveness even when assuming very low drug costs, though its focus on the Swiss health care system makes direct comparison to our US-based analysis difficult. This disagreement is most likely due to several methodological differences that were not shared by our source studies. The authors assume that 1) disadherence to statin therapy is high and 2) there is a small but measurable disutility caused by taking medication, and 3) they assessed costs and benefits over 10 years rather than patient lifetime. Each of these factors significantly decreased statin cost-effectiveness in sensitivity analysis. Particularly, while the authors assume 60% disadherence to statin therapy after 3 years [22], only one of the studies included in our analysis factored significant disadherence in its model [13], while the other three assumed 95–100% adherence; this difference would make statin therapy appear more favorable in the studies we analyzed.

The methodological differences among our source studies might limit direct comparison of their results. Comparing statins to a baseline therapy such as aspirin or diet might make statins appear less cost-effective if the baseline therapy is efficacious and relatively cheap, since in comparison statins will prevent relatively fewer cardiac events for the same cost. Measuring costs and benefits over a longer time period may improve cost-effectiveness, because life-years saved will have a greater chance to accrue, especially for younger populations. Measurement of QALY instead of LYG may be expected to reduce cost-effectiveness, since elderly patients, for whom statins will prevent the most cardiac events, are usually assumed to have a lower quality of life due to disability.

In summary, we found a strong correlation between drug price and cost-effectiveness for healthy patients at varying risk for coronary disease. Statin therapy should be increasingly more cost-effective for lower-risk patients as drug prices decline. Based on cost-effectiveness, it would be reasonable for clinicians to be more aggressive in treating low-risk patients, and for future treatment guidelines to consider recommending therapy for a broader patient base.

## Abbreviations

LDL: Low density lipoprotein; ICER: Incremental cost-effectiveness ratio; CEA: Cost-effectiveness analysis; QALY: Quality adjusted life year; LYG: Life year gained.

## Competing interests

APM: none

RJS: *Consultancies*: (Merck, Pfizer, Liposcience); *Speaking engagements*: (Merck, Pfizer); *Salary support*: (Carolina Center for Medical Excellence); *Research grants*: (Merck)

## Authors’ contributions

APM: Conceived of the study, conducted literature review and statistical analysis, and drafted the manuscript. RJS: Guided study design and scope, revised and edited the manuscript. All authors read and approved the final manuscript.

## Supplementary Material

Additional file 1 Additional details of data extraction from source studies. Click here for file
