# Supplementary material for: Statin cost effectiveness in primary prevention: A systematic review of the recent cost-effectiveness literature in the United States
Source: BMC Res Notes. 2012 Jul 24;5:373. doi: 10.1186/1756-0500-5-373 (PMC3444438; doi:10.1186/1756-0500-5-373)
Supplement: Additional file 1 — Additional details of data extraction from source studies. [file 1756-0500-5-373-S1.doc]

Supplemental Materials

Detailed descriptions of our methods for analyzing data from each study are included here.

*Prosser et al, 2000*

Treatment cost was determined by averaging the stated costs of $1,512 for the first year and $1,318 for all subsequent years over the 30-year time horizon of the study, and then adding the mean of the range of patient time costs as described by the authors. The resulting figure was $1,324.47 per year. The annual drug cost was determined by subtracting from this figure the ancillary costs of physician visits and laboratory testing, again averaged over the 30-year time frame. The resulting value for average annual drug cost was $1,230.70. These values, in 1997 dollars, were then adjusted to 2006 dollars using the consumer price index for medical care.

Data for cost-effectiveness analysis were taken from Table 3, *Cost-Effectiveness of Primary Prevention with a Statin in Men*. In this figure, QALY values were paired with their respective demographic groups. The demographic data was used to determine 10-year Framingham risk levels as calculated by the ATP-III calculator (cite). However, certain data necessary for Framingham risk stratification (total cholesterol and systolic blood pressure) were not included; in these cases, these values were varied within the limits set by the known risk factors (age, diastolic blood pressure, LDL cholesterol, and HDL cholesterol), and the median 10-year risk over the resulting range was used in our analysis. Patients were assumed not to be on medication for blood pressure control. We analyzed data for the 45-54 year age range, and thus used an age of 50 in generating Framingham risk levels.

For generation of data points, we took the mean cost per QALY of all demographic groups falling within one-and-a-half percentage points of the desired Framingham risk level. For example, the cost per QALY at the 10% risk level was determined by averaging the respective values for patient groups falling between 8.5% and 11.5% 10-year risks. For example, the cost per QALY at the 15% risk level was determined by averaging the respective values for patient groups falling between 13.5% and 16.5% 10-year risks.

*Caro et al, 2003*

Annual preventive treatment costs were estimated from the 5-year treatment costs presented in Table 3 by dividing by 5 the difference in total treatment costs for treated patients minus costs for untreated patients in the lowest risk group, resulting in a value of $865.20. Drug cost was determined by subtracting from this value the cost of biannual physician visits and lipid panels, as provided by the authors, giving a drug cost of $791.67 in 1998 dollars.

Costs per LYG for patients at varying 10-year risk levels were also obtained from Table 3; the described 7.8%, 9.8%, 14.4%, and 23.4% risk levels were approximated as 7.5%, 10%, 15%, and 25%, respectively.

*Pignone et al, 2006*

Annual drug cost was $713, as stated in Table 1. Total treatment costs were determined by adding to the additional costs of two office visits, two liver function panels, and one serum lipid panel for patients on statins, giving a value of $839.54.

Costs per QALY were taken from Table 3, at the Framingham risk levels stated therein. Cost per QALY was taken from the row “combination therapy with aspirin and a statin vs. aspirin alone.” Additionally, using Figure 3, the ICERs for patients at 7.5% 10-year risk and annual drug costs of $365 and $1,460 were estimated at $30,000 and $110,000, respectively.

*Pletcher et al, 2009*

Statin costs were $2.11 per day ($770.15 per year) as stated by the authors, with total treatment costs, including an additional office visit and lipid panel annually, of $865.65, in 2006 dollars.

Costs per QALY were taken from Table 2. For each level of cardiac risk, we used the ICER resulting from extending statin therapy to all patients at or above that risk level, as compared with baseline treatment strategies.

For additional data points, we also used data presented in Table 4. Here, additional ICERs for treating patients above 5% and 15% risk were presented relative to varying baseline drug costs.
